# Recent Perspectives on the Pharmacological, Nutraceutical, Functional, and Therapeutic Properties of *Moringa oleifera* Plant

**DOI:** 10.1002/fsn3.70134

**Published:** 2025-04-16

**Authors:** Muhammad Tayyab Arshad, Sammra Maqsood, Ali Ikram, Kodjo Théodore Gnedeka

**Affiliations:** ^1^ University Institute of Food Science and Technology The University of Lahore Lahore Pakistan; ^2^ National Institute of Food Science and Technology University of Agriculture Faisalabad Faisalabad Pakistan; ^3^ Togo Laboratory: Applied Agricultural Economics Research Team (ERE2A) University of Lomé Lomé Togo

**Keywords:** antioxidant, diet, healthy food, tree

## Abstract

The “miracle tree,” *Moringa oleifera* , has gained so much attention because of its spectacular nutritional profile and potential health benefits, making it one of the candidates for functional food product creation. The health benefits of *M. oleifera* are presented here in this review alongside its antioxidant conformation, bioactive constituents, and macro‐ and micronutrient conformation. Moringa is rapidly becoming a key and effective component in formulating healthy foodstuffs because of its outstanding anti‐inflammatory, antimicrobial, antidiabetic, antioxidant, and neuroprotective activities. Investigation into the usage of moringa products in functional foods like dairy substitutes, powders, supplements, drinks, and even snacks is also part of the review. Notwithstanding the auspicious benefits, there are also contests with product development such as issues with sensory perception, active ingredient constancy, and budget constraints. Maintainable plant‐based and healthy food demand across the sphere is pushing the novelty in the moringa industry. To improve the current contests and the scalability and suitability of moringa‐based functional foods, this review concludes with a call for additional investigation.

## Graphical abstract

Despite the remarkable potential of moringa, we also identify significant challenges in product development, such as sensory perception issues, ingredient stability, and financial constraints. Given the increasing global demand for plant‐based, sustainable, and health‐conscious meals, we emphasize the need for continued innovation in moringa‐based food products. The review concludes with a call for further research to address these challenges and enhance moringa's scalability and consumer acceptance in the food industry.

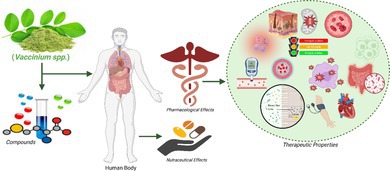

## 1 Introduction

The establishment of nutrients, functional foods can also avoid or improve health by comprising bioactive ingredients, vitamins, minerals, or phytochemicals (Aghajanpour et al. 2017). Because of their capability to alleviate long‐lasting diseases such as diabetes, cancer, and cardiovascular complications through mechanisms such as antioxidant activity, anti‐inflammatory properties, and immunomodulation, the foods have attracted a great deal of interest (Sharma and Yadav 2022). With the eventual vision of ornamental public health, functional foods frequently contain naturally rich nutrient substitutes, dietary supplements, and fortified foods. Personalized nutrition, which has been attracting more attention recently, highlights their role in improving dietary consequences between different groups (Gul et al. 2016). The integration of functional foods in consistent meals eases overall well‐being by satisfying the gap between nutrition and medicine (Anil et al. 2022). Developments in nutrigenomics and food technology have amended the identification and application of bioactive constituents to deliver solutions for disease deterrence and health elevation (Boopathi and Raveendran 2021).

For instance, *M. oleifera* is one of the best examples of the transformative potential of functional foods, especially in addressing oxidative stress and malnutrition, because of its rich profile of vitamins, minerals, and bioactive phytochemicals (Popoola and Obembe 2013; Gandji, Chadare, et al. 2018; Gandji, Salako, et al. 2018). As suggested by continued research, functional foods might help improve well‐being as they could simultaneously tackle the twin issues of developed and developing countries related to high chronic disease prevalence and nutrient deficiencies (Dixit et al. 2016; Gupta et al. 2018).

*Moringa oleifera* is produced under variable circumstances and fabrication practices in Pakistan and Togo. The biological conformation and antioxidant capability of the leaves of Pakistani Moringa are prejudiced by geography and season (Iqbal and Bhanger 2006). The agronomic and financial implications of the Pakistani Moringa landraces have been emphasized by an outcome that they can produce premium oil (Faisal et al. 2020). Plantation circumstances disturb Moringa growth and production in Togo, where it is rummaged extensively in agroforestry (Abotsi et al. 2015). El Bilali et al. (2024) quote other African studies that validate Moringa's significance in food security and sustainable farming.

The “drumstick tree” or “miracle tree” *M. oleifera* has been widely rummaged in sustainable agriculture and medicine for thousands of years. Factually, its usage was renowned in the foothills of the Himalayan area of India for the first time. Gandji, Chadare, et al. (2018) and Gandji, Salako, et al. (2018) identified that the plant presently encompasses much of the planet, comprising subtropical and tropical states in Latin America, Africa, and Asia. Its usage as a food element, medicine, and even a water purifying mediator defends its importance within the cultural lives of maximum societies (Gupta et al. 2018; Rohim et al. 2024). Its capability to stand diverse climatic circumstances and resilience in arid and semi‐arid parts exemplifies its worth toward food security as well as conservation of the atmosphere (Lakshmidevamma et al. 2021; Saras 2023). MO was rummaged to treat over 300 illnesses, as mentioned in the ancient Ayurvedic scriptures (Dixit et al. 2016).

Its solicitation in African traditional medication and food, particularly in regions where hunger is a problem, designates that *M. oleifera* 's cultural value spreads beyond India (Matic et al. 2018). The tree is also identified as “Garkee” in Nigeria, for occurrence. Due to its medicinal possessions and prospective to earn money from its leaves, seeds, and oil, it is appreciated for its socioeconomic welfares (Popoola and Obembe 2013). Moreover, the farming of Moringa in Benin is straightly affected by the presence of fertile land and proximity to marketplaces thereby affecting its solicitation in farming and medicine (Gandji, Chadare, et al. 2018; Gandji, Salako, et al. 2018). The cultural significance of Moringa is also reproduced in its operation as a basic food. Its leaves, fruits, and seeds are precious components of traditional cuisine due to their influence of perilous amino acids, calcium, potassium, and vitamins A, C, and E (Alegbeleye 2018; Trigo et al. 2020). Its leaves are typically consumed as a vegetable or used to enhance flavor to soups and sauces in maximum African and Southeast Asian nations (Liu et al. 2018; Nathaniel et al. 2020). Furthermore, its therapeutic solicitations against inflammation, diabetes and malnutrition, between others, have defensible this species' significance in traditional medicine (Hedhili et al. 2022; Peñalver et al. 2022).

Over the past years, *M. oleifera* 's worldwide appeal as a “superfood” has also strengthened its cultural and historical importance. Western countries have begun to investigate its potential as a functional food, making use of its bioactive constituents to yield health foods and nutraceuticals (Yang et al. 2023). The ecological benefits of this plant, comprising increasing soil quality and acting as a carbon sink, mark it as further vital in climate variation planning (Boopathi and Raveendran 2021). As a custodian of cultural heritage and an embodiment of transformation, *M. oleifera* is a moral example of this assembly of traditional knowledge and modern health investigation, where tradition and newly developed solicitations still meet (Chhikara et al. 2021; Kashyap et al. 2022). Thus, aside from being an association among nature and nutrition, *M. oleifera* is a poignant notice of the deep interface of ancient traditions with contemporary usages towards improving worldwide sustainability and health (Dzuvor et al. 2022; Mushtaq et al. 2021). Its prominence will continue to rise as its supreme potential is acknowledged by communities as well as researchers, certifying this “miracle tree” keeps changing lives all over the world.

This review purposes to deliberate the nutritional value and numerous health compensations of *M. oleifera* , with a specific emphasis on its convention in functional foods. Through these, it looks into its beneficial and adaptive incorporation as an adjunct to promote health through rich nutrition, including key macro‐ and micronutrients and its potent bioactive compounds. It explores the many health advantages, from cardiovascular, neurological, and immune‐boosting impacts to antioxidant and anti‐inflammatory qualities. The review explores its uses in producing functional foods such as powders, beverages, baked goods, and culinary products.

## 2 Nutritional Composition of *Moringa oleifera*

*Moringa oleifera* has interested nearly everybody, even from other parts of the globe, with very nutri luxuriant riches, particularly macronutrient richness like protein, dietary fiber, and fat reserves. Being composed as it is, it represents a rich tool for treating deficiencies in the diet and improving general well‐being, particularly when malnutrition is commonplace. *M. oleifera* is essential as a dietary component due to the substantial plant‐based protein it provides, especially for populations with a limited animal protein intake. It has leaves with an extremely high protein content and a balanced range of amino acids necessary to the human body, which the latter cannot produce on its own (Table 1) (Yang et al. 2023).

**TABLE 1:** Nutritional composition of *Moringa oleifera* .

Component	Details	References
Macronutrients	High in protein (25%–27%), fiber (13%–16%), and healthy fats (1%–3%)	Yang et al. (2023), Kashyap et al. (2022), Arora and Arora (2021), Ntshambiwa et al. (2023)
Vitamins	Rich in Vitamin A (beta‐carotene), B‐complex (B1, B2, B3), C, and E	Sultana (2020), Guzmán‐Maldonado et al. (2020), Olusanya et al. (2020), Hadju et al. (2021)
Minerals	Contains calcium, potassium, iron, zinc, and magnesium	Masitlha et al. (2024), Zungu et al. (2020), Kamran et al. (2020), Nuapia et al. (2020)
Polyphenols	Includes quercetin, kaempferol, and chlorogenic acid	Khalid et al. (2023), Peñalver et al. (2022), Chhikara et al. (2021)
Flavonoids	Luteolin, apigenin, and rutin prevalent	Dzuvor et al. (2022), Kashyap et al. (2022), Calizaya‐Milla et al. (2022)
Isothiocyanates	Significant in glucosinolates‐derived bioactive compounds	Ntshambiwa et al. (2023), Yang et al. (2023), Mushtaq et al. (2021)
Glucosinolates	Notable for health‐promoting properties; supports anticancer mechanisms	Khalid et al. (2023), Ntshambiwa et al. (2023), Kashyap et al. (2022)
Alkaloids	Contains alkaloids like moringine with therapeutic potential	Olvera‐Aguirre et al. (2022), Dzuvor et al. (2022)
Antioxidants	High antioxidant capacity due to phenolics and Vitamin C	Kamran et al. (2020), Hadju et al. (2021), Khalid et al. (2023)
Carotenoids	Rich in beta‐carotene, lutein, and zeaxanthin	Sultana (2020), Zungu et al. (2020), Chhikara et al. (2021)
Other Bioactives	Saponins, tannins, and terpenoids present	Kashyap et al. (2022), Dzuvor et al. (2022), Guzmán‐Maldonado et al. (2020)
Dietary Applications	Fortified in snacks, beverages, and complementary foods for improving nutrient profiles	Arora and Arora (2021), Nathaniel et al. (2020), Roni et al. (2021), Masitlha et al. (2024)

This protein profile strictly adheres to WHO's standards, enhancing its potency against protein‐energy malnutrition (Kashyap et al. 2022). In accumulation, its amino acid profile with leucine, isoleucine, and valine supports muscle reparation and development, hence its appropriateness for athletes and rehabilitation patients (Ntshambiwa et al. 2023). *M. oleifera* was exploited as a protein supplement in the expansion of functional foods like protein bars, powders, and fortified snacks. These revolutions exploit the high protein bioavailability of the plant to gratify nutritional requirements across diverse populations such as children and the elderly (Liang et al. 2023; Arora and Arora 2021).

Another vigorous fundamental of *M. oleifera* is dietary fiber. The mainstay of a healthy gut is fiber, and moringa fiber controls bowel movements and averts ailments such as constipation and irritable bowel syndrome (Sultana 2020). Dietary fiber decreases the absorption rate of glucose, improving glycemic control, thus creating it appropriate for diabetic patients or even those predisposed to emerging the illness (Peñalver et al. 2022). The fiber in the leaf of *M. oleifera* also assists in upholding cardiovascular health through the sequestration of cholesterol and easing its excretion. The regular usage of high‐fiber foods like moringa reduces levels of LDL cholesterol and risk influences for heart illness (Yang et al. 2023). Through the conquest of hunger and consumption of fewer total calories, the satiety consequence of fiber also assists in weight loss (Kashyap et al. 2022).

Though the leaves of *M. oleifera* might not importantly raise the inclusive fat content, trace quantities of convenient lipids like omega‐3 and omega‐6 fatty acids are present in them. Ntshambiwa et al. (2023) state that these lipids improve the plant's anti‐inflammatory and cardioprotective effects which have a confident association with overall health. Certain of the other nutritional compensations of moringa's unsaturated fatty acids comprise better skin health, stability of hormones, and mental function (Arora and Arora 2021). *M. oleifera* is a full nutritional supplement as it is rich in lipids, fiber, and protein. The mass manufacture of its leaves into powders and other foodstuffs such as soups, smoothies, and herbal drinks has rendered its nutritional compensations available to the community (Sultana 2020). Due to these physiognomies, *M. oleifera* is an operative solution for nutritional deficits and an essential part of functional diets.

*Moringa oleifera* owns abundant nutritional and useful benefits; it is an outstanding source of carbohydrates that can be expended and comprises high energy content. Their leaves are much extolled for being rich in protein and fiber content, though general nutritional value is basically based on carbohydrates. Most of the multifaceted carbohydrates in *M. oleifera* leaves produce energy slowly ensuing digestion (Wang et al. 2024). These carbohydrates are significant to deliver the body with the daily energy required, particularly in resource‐scarce surroundings where the energy content of staple foods might be squat (Yang et al. 2023). In order to provide continuing energy, leaves are frequently expended in diets, particularly in populations at risk of undernutrition (Sultana 2020). Moreover, the carbohydrate content contains both soluble and insoluble fiber that endorses intestinal health and a normal glycemic reaction (Kashyap et al. 2022).

The prevalent tree *M. oleifera* is also high in proteins, carbs, and vitamins and minerals. The micronutrients liable for building the tree such a well‐known “superfood” comprise many nutrients, and they are ironic in numerous health benefits. Certain of the numerous vitamins in *M. oleifera* leaves comprise vitamin A, B‐complex vitamins, vitamin C, and vitamin *E. beta* ‐carotene, existing in moringa leaves, is a vitamin A precursor and is desirable for immune function, eye health, and skin health. Cell growth, communication, and reproduction also necessitate vitamin A. This source of vitamin is controlled in moringa leaves and is supposed to be important in their antioxidant possessions (Yang et al. 2023; Peñalver et al. 2022). The B vitamins present in moringa comprise folate, B12, B1 (thiamine), B2 (riboflavin), B3 (niacin), and B6 (pyridoxine). These B vitamins are complicated in energy metabolism, the amalgamation of red blood cells, and brain function. For example, vitamin B6 is critical in protein metabolism and mental function, while folate is vigorous for DNA reparation and synthesis (Kashyap et al. 2022).

Vitamin C, a vitamin that endorses immunity, iron preoccupation, and production of collagen for healthy skin, is foremost profuse in moringa leaves. Due to the occurrence of this vitamin, moringa is an extremely noteworthy meal for improving immunity and anticipation in contradiction of scurvy disorders (Sultana 2020; Yang et al. 2023). Vitamin E, a plant‐based antioxidant, defends human cells from oxidative stress and harm caused by free radicals. This vitamin is liable for healthy skin and reproductive health (Yang et al. 2023). *M. oleifera* comprises a very great mineral content. The most predominant minerals are calcium, potassium, iron, and zinc, which are all recognized to be essential for numerous functions in the body.

*Moringa oleifera* leaves comprise high levels of calcium, which is compulsory for bone and muscle strength. It enables the secretion of hormones and nerve transmission. This diet is better when people expect plant‐based sources of calcium because moringa comprises more minerals associated with most of the traditional sources, including milk (Masitlha et al. 2024; Yang et al. 2023). Potassium, a key electrolyte involved in heart well‐being, muscle shrinking, and hydration, is found in copious quantities in moringa leaves. It also maintains sound brain function and blood pressure variation (Peñalver et al. 2022). An additional mineral within moringa plants is iron, which assists in making hemoglobin in red blood cells and blood flow through the body. Anemia, predominant in most areas of the ecosphere, results from iron deficiency, which moringa has been found to treat (Yang et al. 2023; Sultana 2020). Zinc plays a role in immune function, wound healing, and protein metabolism. Moringa leaves are rich in zinc, as found by Kashyap et al. (2022) and Peñalver et al. (2022), and provide growth and development, particularly in children and pregnant women. In addition, it conserves the health of the skin and augments immune function. One of the foods that is tremendously rich in essential vitamins and minerals, *M. oleifera* is very densely packed with nutrients. Whereas vitamins A, B‐complex, C, and E have antioxidant and immunological properties, calcium, potassium, iron, and zinc uphold the health of bones, metabolism, and general welfare. The health of most individuals can be improved, and vitamin deficiencies can be significantly reduced by including moringa in their diet, particularly in emerging countries with limited resources (Table 2) (Yang et al. 2023; Sultana 2020; Kashyap et al. 2022).

**TABLE 2:** Composition of *Moringa oleifera* leaves.

Nutrient	Fresh leaves (per 100 g)	Leaf powder (per 100 g)	Processing method	References
Moisture	75–80 g	5–7 g	Sun‐dried, powdered	Olson et al. (2016), Singh and Prasad (2013)
Protein	6–9 g	25–30 g	Blanched, dried, powdered	Biel et al. (2017), Kashyap et al. (2022)
Fat	1–2 g	5–7 g	Sun‐dried, powdered	Ikram et al. (2023), Vergara‐Jimenez et al. (2017)
Carbohydrates	12–15 g	35–40 g	Blanched, dried, powdered	Olson et al. (2016), Singh and Prasad (2013)
Fiber	2–3 g	10–12 g	Sun‐dried, powdered	Biel et al. (2017), Kashyap et al. (2022)
Vitamin C	120–150 mg	15–20 mg	Blanched, dried, powdered	Olson et al. (2016), Singh and Prasad (2013)
Vitamin A (β‐carotene)	6–8 mg	15–20 mg	Sun‐dried, powdered	Biel et al. (2017), Kashyap et al. (2022)
Calcium	200–250 mg	2000–2500 mg	Blanched, dried, powdered	Olson et al. (2016), Singh and Prasad (2013)
Iron	2–3 mg	25–30 mg	Sun‐dried, powdered	Biel et al. (2017), Kashyap et al. (2022)
Potassium	250–300 mg	1300–1500 mg	Blanched, dried, powdered	Olson et al. (2016), Singh and Prasad (2013)
Magnesium	40–50 mg	350–400 mg	Sun‐dried, powdered	Biel et al. (2017), Kashyap et al. (2022)
Zinc	0.5–1 mg	3–4 mg	Blanched, dried, powdered	Olson et al. (2016), Singh and Prasad (2013)
Phosphorus	50–60 mg	200–250 mg	Sun‐dried, powdered	Biel et al. (2017), Kashyap et al. (2022)
Antioxidants (Total phenolics)	100–150 mg GAE	300–400 mg GAE	Blanched, dried, powdered	Olvera‐Aguirre et al. (2022), Basharat et al. (2023)
Flavonoids	20–30 mg QE	50–70 mg QE	Sun‐dried, powdered	Olvera‐Aguirre et al. (2022), Basharat et al. (2023)

Abbreviations: GAE, gallic acid equivalents; QE, quercetin equivalents.

### 2.1 Bioactive Profile of *Moringa oleifera*

**FIGURE 1:**
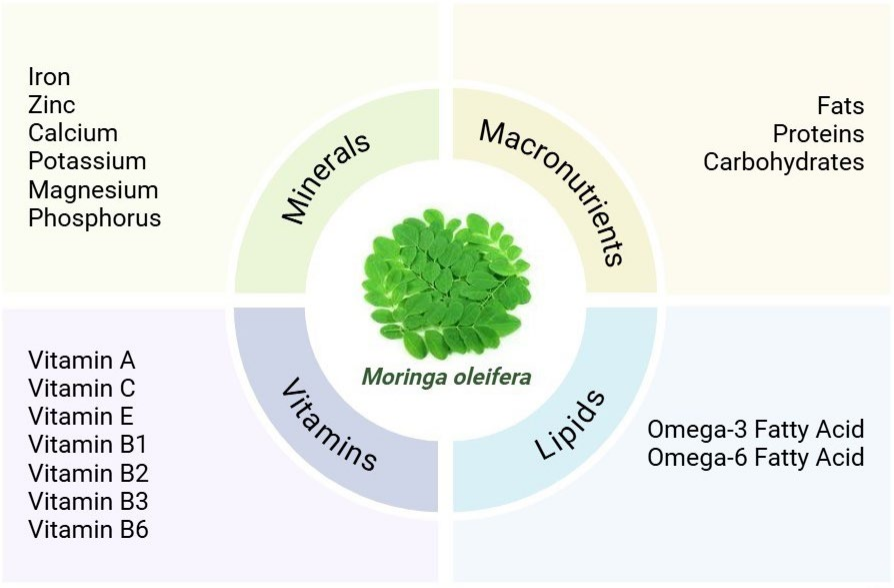
Composition of *Moringa oleifera* .

*Moringa oleifera* is commonly known as the “drumstick tree” owing to its high diversity in bioactive substances that facilitate many health benefits. It includes polyphenols, flavonoids, isothiocyanates, glucosinolates, and alkaloids with potent anti‐inflammatory, anticancer, and antioxidant properties (Figure 1).

#### 2.1.1 Polyphenols

There are vast classifications of naturally occurring chemicals having antioxidant properties, such as polyphenols (Li et al. 2024). Evidence proves these leaves in *M. oleifera* have been shown to decrease oxidative stress within the human body, resulting in avoiding chronic diseases, for instance, cancer and heart‐related issues (Owon et al. 2021). In addition, it can prevent cellular damage while it inhibits inflammation as well.

#### 2.1.2 Flavonoids

Another set of compounds in *M. oleifera* , with well‐known anti‐inflammatory and antioxidant characteristics, includes flavonoids. The given agents help protect cells against inflammatory and oxidative damage—two crucial drivers of the onset of a number of degenerative conditions. Diabetic patients could, therefore, derive a value from their flavonoids, which have antibacterial activity that makes it possible to lower blood glucose (Olusanya et al. 2020).

#### 2.1.3 Isothiocyanates

Isothiocyanates, sulfur‐containing compounds, have been reported to be associated with cancer. These compounds in moringa facilitate detoxification by the body, and the procedure will halt the formation of cancerous cells. Besides, they help the liver perform detoxification processes, which enhance the body's ability to get rid of toxins (Kashyap et al. 2022).

#### 2.1.4 Glucosinolates

The second category of sulfur compounds in *M. oleifera* includes glucosinolates. Their capability to interfere with detoxifying and cell cycle‐related enzymes made them responsible for inhibiting cancer development. It was observed that glucosinolates in moringa elevated immunity and might even inhibit inflammatory responses within the body (Shang et al. 2024; Chhikara et al. 2021).

#### 2.1.5 Alkaloids

Nitrogenous compounds, known as alkaloids, show various types of medicinal potential, which include analgesic, antibacterial, and anti‐inflammatory effects. One of the explanations moringa is capable of assisting with pain, sleep, and general well‐being is due to its alkaloids (Roni et al. 2021). Such chemicals augment the therapeutic activity of moringa leaves in contradiction to a number of illnesses owing to their extraordinary biological activity.

**FIGURE 2:**
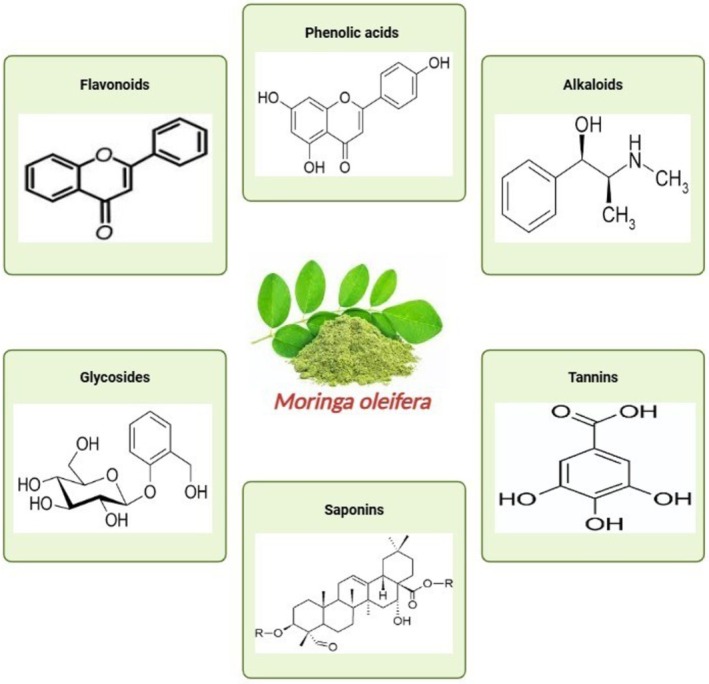
Chemical structures of phytochemicals in *Moringa oleifera* .

Last but not least, *M. oleifera* is antioxidant, anti‐inflammatory, and anticancer in nature as a consequence of its comprehensive arsenal of bioactive composites, among which are polyphenols, flavonoids, isothiocyanates, glucosinolates, and alkaloids. Frequent chronic illnesses and disorders such as cancer, cardiovascular disease, and neurological injury are caused by free radicals, against which *M. oleifera* can scavenge. This is because of its antioxidant capability (Hu et al. 2023; Guzmán‐Maldonado et al. 2020). Antioxidant scavenging activity is meaningfully augmented by bioactive constituents present in moringa, comprising polyphenols, flavonoids, and isothiocyanates (Figure 2). They constrain oxidative stress and free radical impairment by donating electrons to the free radicals. Experts have exposed that Moringa leaves comprise strong antioxidant physiognomies because of the high content of their phenolic constituents (Guzmán‐Maldonado et al. 2020; Owon et al. 2021). This is one of the explanations why the plant can be resilient to oxidative stress. Another reason this plant has therapeutic potential is that its antioxidant properties have been linked to its anti‐inflammatory effects because the reduction in oxidative stress also reduces inflammation (Table 3) (Kashyap et al. 2022).

**TABLE 3:** Bioactive compounds in *Moringa oleifera* leaves.

Bioactive compound	Quantity (per 100 g)	Uniqueness	Health benefits	References
Moringin (Isothiocyanate)	5–10 mg	Stable isothiocyanate with 4 classes; activates TRPA1 channels	Anti‐inflammatory, neuroprotective, and anti‐cancer properties	Borgonovo et al. (2020), Kashyap et al. (2022)
Mamuroside A (Alkaloid)	2–5 mg	Unique alkaloid with antioxidant properties	Reduces oxidative stress and inflammation	Kamran et al. (2020), Kashyap et al. (2022)
Mamuroside B (Alkaloid)	1–3 mg	Rare alkaloid with antidiabetic properties	Improves insulin sensitivity and glucose metabolism	Kamran et al. (2020), Kashyap et al. (2022)
Quercetin (Flavonoid)	20–30 mg	Potent antioxidant and anti‐inflammatory flavonoid	Protects against cardiovascular diseases and cancer	Kashyap et al. (2022), Vergara‐Jimenez et al. (2017)
Kaempferol (Flavonoid)	10–15 mg	Antioxidant and anti‐inflammatory flavonoid	Reduces risk of chronic diseases and supports immune function	Kashyap et al. (2022), Vergara‐Jimenez et al. (2017)
Chlorogenic Acid (Phenolic Acid)	50–100 mg	Major phenolic acid with antioxidant properties	Reduces blood pressure and improves glucose metabolism	Kashyap et al. (2022), Olvera‐Aguirre et al. (2022)
Caffeic Acid (Phenolic Acid)	10–20 mg	Antioxidant and anti‐inflammatory phenolic acid	Protects against oxidative stress and supports liver health	Kashyap et al. (2022), Olvera‐Aguirre et al. (2022)
Rutin (Flavonoid)	5–10 mg	Bioflavonoid with strong antioxidant properties	Improves vascular health and reduces inflammation	Kashyap et al. (2022), Vergara‐Jimenez et al. (2017)
β‐Sitosterol (Phytosterol)	10–20 mg	Plant sterol with cholesterol‐lowering properties	Reduces LDL cholesterol and supports heart health	Kashyap et al. (2022), Chhikara et al. (2021)
Gallic Acid (Phenolic Acid)	5–10 mg	Antioxidant and antimicrobial phenolic acid	Protects against infections and oxidative damage	Kashyap et al. (2022), Olvera‐Aguirre et al. (2022)
Epigallocatechin Gallate (EGCG)	2–5 mg	Catechin with strong antioxidant and anti‐cancer properties	Reduces cancer risk and supports brain health	Kashyap et al. (2022), Vergara‐Jimenez et al. (2017)
Zeatin (Cytokinin)	1–2 mg	Plant hormone with anti‐aging properties	Promotes cell growth and delays aging	Kashyap et al. (2022), Chhikara et al. (2021)
Vitamin C (Ascorbic Acid)	120–150 mg	Water‐soluble antioxidant vitamin	Boosts immunity and protects against oxidative stress	Olson et al. (2016), Singh and Prasad (2013), Mohamed et al. (2022)
Vitamin E (Tocopherol)	5–10 mg	Fat‐soluble antioxidant vitamin	Protects cell membranes and reduces oxidative damage	Olson et al. (2016), Singh and Prasad (2013), Rohim et al. (2024)
Carotenoids (β‐Carotene)	6–8 mg	Precursor to vitamin A with antioxidant properties	Supports vision and immune function	Biel et al. (2017), Kashyap et al. (2022), Mahmoud et al. (2024)
Polyphenols (Total)	300–400 mg GAE	Diverse group of antioxidants	Reduces oxidative stress and inflammation; supports heart health	Olvera‐Aguirre et al. (2022), Basharat et al. (2023), Mohamed et al. (2022)
Flavonoids (Total)	50–70 mg QE	Group of antioxidants with anti‐inflammatory properties	Protects against chronic diseases and supports brain health	Olvera‐Aguirre et al. (2022), Basharat et al. (2023), Rohim et al. (2024)
Tannins	10–20 mg	Astringent polyphenols with antimicrobial properties	Protects against infections and supports gut health	Kashyap et al. (2022), Chhikara et al. (2021), Mahmoud et al. (2024)
Saponins	5–10 mg	Natural detergents with cholesterol‐lowering properties	Reduces cholesterol and supports immune function	Kashyap et al. (2022), Chhikara et al. (2021), Mohamed et al. (2022)
Alkaloids (Total)	5–10 mg	Nitrogen‐containing compounds with diverse biological activities	Anti‐inflammatory, analgesic, and anti‐cancer properties	Kamran et al. (2020), Kashyap et al. (2022), Rohim et al. (2024)

Abbreviations: GAE, gallic acid equivalents; QE, quercetin equivalents.

## 3 Pharmacological Properties

### 3.1 Bioactive Compounds and Their Mechanisms of Actions

Among the numerous pharmacological composites present in leaves of *M. oleifera* (MO) are anti‐inflammatory, analgesic, antioxidant, and antibacterial properties. Isothiocyanates, polyphenols, alkaloids, and flavonoids are some of the most famous bioactive composite complexes that affect physiological processes (Luo et al. 2024). Moringin, a stable isothiocyanate isolated from MO, was shown to strongly activate the ankyrin 1 transient receptor potential (TRPA1) channel, which contributes to pain sensations and inflammatory reactions (Borgonovo et al. 2020). Due to this stimulation, nociceptive signals are controlled, thus making MO a good outlook for pain management treatment.

Due to their strong polyphenol and flavonoid configuration, MO leaves are an operative antioxidant when they have accrued. By scavenging reactive oxygen species (ROS), one of the mechanisms through which these composites postpone the development of long‐lasting diseases is illustrated (Olvera‐Aguirre et al. 2022; Vergara‐Jimenez et al. 2017). Additionally, Kashyap et al. (2022) argue that MO polyphenols regulate pro‐inflammatory cytokines such as interleukin‐6 (IL‐6) and tumor necrosis factor‐alpha (TNF‐α). The antibacterial properties of MO leaf have been attributed to its high isothiocyanate and alkaloid content. The composites inhibit bacterial growth by compromising their cell walls, creating MO leaf an effective tool in the fight against foodborne infections (Chhikara et al. 2021). The involved bioactive ingredients of *M. oleifera* leaves have expanded pharmacological attention and have potential as a phytotherapeutic mediator in numerous applications.

#### 3.1.1 Antioxidant Effect

**FIGURE 3:**
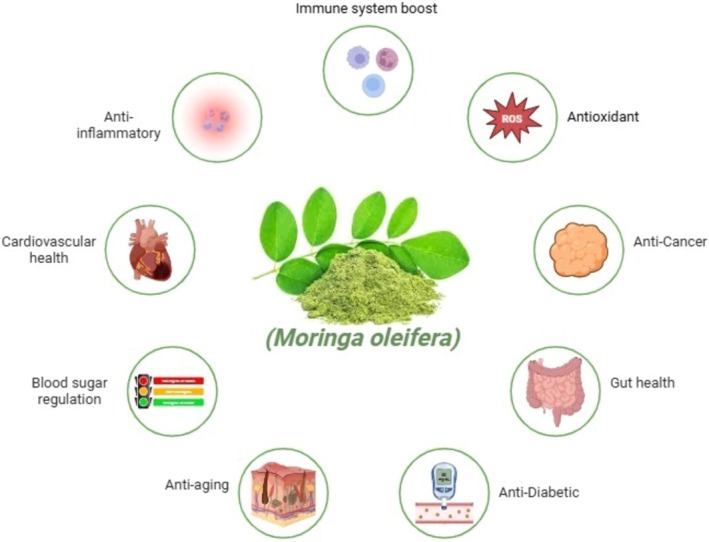
Potential health benefits of *Moringa oleifera* .

Commonly called the “miracle tree,” *M. oleifera* has been the object of many research interests because of its unique nutritional profile and medicinal properties. There are several health benefits to having moringa, but perhaps among its most substantial advantages are its anti‐inflammatory properties and antioxidants (Pop et al. 2022). The pathophysiology of most chronic diseases, including diabetes, cancer, cardiovascular disorders, and neurodegenerative diseases, is mainly caused by oxidative stress and chronic inflammation, which these properties are critical in preventing (Ramamurthy et al. 2021; Avilés‐Gaxiola et al. 2021). These medicinal properties are primarily based on the bioactive compounds present in moringa, such as polyphenols, flavonoids, glucosinolates, and isothiocyanates. Numerous disorders are connected to oxidative stress, which ascends when reactive oxygen species (ROS) in the blood are out of balance with the antioxidant defense the body has. Antioxidants are cooperative as they find homeostasis, scavenge free radicals, and preserve cells from injury. *M. oleifera* has high antioxidant activity because of its varied bioactive compounds. The seeds, leaves, and pods of the moringa plant comprise beta‐carotene, vitamin C, ascorbic acid, phenolic acids, and flavonoids. Ramamurthy et al. (2021) and Avilés‐Gaxiola et al. (2021) have stated that such substances permit the plant to scavenge free radicals. In vitro studies have shown that the strong antioxidant activity of Moringa leaf extracts has a close connection with their phenolic content. Quercetin and kaempferol are two free radical scavenging polyphenols that efficiently prevent oxidative impairment to proteins, lipids, and DNA (Varadarajan and Balaji 2022). These compounds reduce oxidative stress and fortify the body's antioxidant protection system by increasing the activity of antioxidants like superoxide dismutase and catalase (Avilés‐Gaxiola et al. 2021). Furthermore, investigations have established that moringa is capable of averting or postponing the onset of oxidative stress‐associated illnesses owing to its antioxidant properties. Animal investigations have established that Moringa extracts can improve antioxidant enzyme activity as well as decrease levels of lipid peroxidation, which are markers for oxidative impairment (Mthiyane et al. 2022). This designates that moringa has the potential to avert diseases such as diabetes, cancer, and atherosclerosis by lowering oxidative impairment levels (Figure 3).

#### 3.1.2 Anti‐Inflammatory Effect

All classes of diseases fluctuating from diabetes to cardiovascular disease to arthritis to neurological disorders have chronic inflammation as one of their reasons. The extensive usage of *M. oleifera* by the traditional medicinal system to treat inflammation has been authorized by current studies. The anti‐inflammatory properties of moringa result from its capability to overpower the activation of compounds that are responsible for inflammation, such as cytokines, cyclooxygenase (COX‐2) and nuclear factor kappa light chain enhancer of activated B cells (NF‐κB), according to Saleem et al. (2020) and Ramamurthy et al. (2021). Critical in moderating inflammatory reactions are the bioactive compounds of moringa, which comprise the flavonoids kaempferol and quercetin. The amalgamation of pro‐inflammatory cytokines, such as TNF‐α and IL‐1, and IL‐6 frequently raised in chronic inflammatory illnesses, is repressed by these compounds (Saleem et al. 2020). In addition, studies have confirmed that Moringa extracts overpower the activation of NF‐κB, a transcription factor modifying the expression of numerous genes involved in inflammation. This activity is critical for reducing systemic inflammation, a representative of inflammatory bowel disease, rheumatoid arthritis, and neurological illnesses such as Alzheimer's, according to Mairuae et al. (2023). In addition to its influence on systemic inflammation, moringa also has anti‐inflammatory properties when used topically.

Inflammatory skin conditions comprising eczema and psoriasis have been relieved by dermatologists with the usage of Moringa seed oil. Through the decrease of hyperproliferation and inflammation, the oil is a prospective substitute treatment for inflammatory skin diseases (Cretella et al. 2020). Due to its antioxidant and anti‐inflammatory properties, *M. oleifera* is an actual treatment option for illnesses categorized by oxidative stress and chronic inflammation. In diabetes, for example, high blood glucose levels induce oxidative stress and the cohort of free radicals; these in turn trigger inflammatory reactions that aggravate insulin resistance (Xiong et al. 2022). Due to its anti‐inflammatory and free radical scavenging activities, moringa could be helpful in the management of diabetes. Methodical studies have established that moringa extracts were capable of reducing blood glucose concentration and inflammatory cytokines in diabetic rats induced with streptozotocin (Mthiyane et al. 2022). This suggests that the extracts have the potential of being an effective natural medication against diabetes. Also, oxidative stress and inflammation play vital parts in the causation of neurologic illnesses such as Alzheimer's and Parkinson's. It is supposed that the antioxidant and anti‐inflammatory properties of moringa are involved in its neuroprotective role.

Inhibition of neuroinflammatory procedures and antioxidant decrease of oxidative impairment in neuronal cells have been shown by moringa plant extracts. Shahbaz et al. (2024) elucidate that these procedures can be useful in the defense of neurons and cognitive functions and may be useful as a cure for neurodegenerative illnesses. The ironic content of antioxidant and anti‐inflammatory ingredients in *M. oleifera* has an important influence in decreasing oxidative stress and long‐lasting inflammation, which reasons numerous health benefits. Flavonoids, polyphenols, and isothiocyanates are just specific examples of the plant's bioactive composites that are acute to free radical scavenging, variation of the inflammation path, and evasion of cell damage. Moringa can decrease inflammation and oxidative stress and thus develops a probable therapeutic mediator for numerous chronic diseases. They comprise diabetes, cardiovascular disease, cancer, and neurological sicknesses. Further investigation on the health benefits and illness prevention competencies of moringa would clarify its role.

#### 3.1.3 Cardioprotective Effect

The drumstick tree, also mentioned as *M. oleifera* , is a plant that has expanded in popularity due to its unbelievable medicinal properties, one of which is its capability to benefit the heart. Due to the noteworthy role played by the cardiovascular system in general health, cardiovascular ailments like hypertension, atherosclerosis, and heart attacks are specific as the leading reasons for death and disability internationally. The bioactive compounds of *M. oleifera* have been shown to recover cardiovascular health through the decrease of blood pressure and cholesterol. The substance consists of polyphenols, flavonoids, vitamins, and minerals. The antioxidant, anti‐inflammatory, and lipid‐lowering properties of the plant are largely accountable for its benefits, most of which are associated with improved cardiovascular health. Central to cardiovascular health is that *M. oleifera* decreases levels of cholesterol, particularly “bad” cholesterol LDL Atherosclerosis causes heart attacks and strokes, and high LDL cholesterol is a main risk factor for this disease. Moringa leaf extracts upsurge levels of “good” HDL cholesterol while significantly decreasing “bad” LDL and total cholesterol, as per studies (Chhikara et al. 2021; Randriamboavonjy et al. 2016). Supplementation with Moringa, one of the vital constituents in the prevention of cardiovascular disorders, has caused diminished blood cholesterol levels in hyperlipidemic rats (Kashyap et al. 2022). Meanwhile, they constrain cholesterol synthesis and improve its breakdown; polyphenolic compounds such as quercetin and chlorogenic acid present in Moringa leaves are most likely to be responsible for this cholesterol‐lowering activity (Camilleri and Blundell 2024).

The solicitation of *M. oleifera* to accomplish hypertension has also revealed promising results. The conservation of a healthy heart necessitates the continuous monitoring of blood pressure and its treatment as hypertension (elevated blood pressure) is one of the main risk factors for cardiovascular disorders. Numerous studies have shown that moringa is proficient in augmenting cardiovascular well‐being by dropping systolic and diastolic blood pressure levels. The antihypertensive action of the material is postulated by Alia et al. (2022) to be affected by its rich potassium, magnesium, and calcium conformation. These reasons contribute to the proper function of the capillaries, which decreases blood pressure through reduced vascular resistance. The antioxidant properties of moringa contribute to lessening oxidative stress, which further constrains endothelial dysfunction and hypertension (Hassan et al. 2021). By enhancing the activity of endothelial nitric oxide synthase, animal prototypes have revealed that moringa supplementation decreases blood pressure.

As per Wal et al. (2024), this enzyme contributes to vasodilation and diminished vascular tension through the cohort of nitric oxide. *M. oleifera* has revealed promise in directly supporting the heart and refining overall cardiovascular health alongside its effect on blood pressure regulation and cholesterol reduction. Plant antioxidants such as ascorbic acid, flavonoids, and phenolics contribute to neutralizing free radicals and reducing the impairment that oxidative stress can inflict on cardiac tissues (Camilleri and Blundell 2024). Persistent oxidative stress is one of the leading causes of heart disease, but moringa can protect cardiac cells and tissues against this danger. Another reason moringa extracts are helpful for blood vessels is that they decrease inflammation, and inflammation is one of the key risk factors for cardiovascular disease. Atherosclerosis can be mitigated by the anti‐inflammatory properties of moringa, which dampen the inflammatory reaction and thus the formation of arterial plaque that follows (Alia et al. 2022). Randriamboavonjy et al. (2016) exposed that Moringa seed oil protects spontaneously hypertensive rats from cardiac hypertrophy, a condition where there is an irregular thickening and reduced functioning of heart muscle from occurring.

#### 3.1.4 Antidiabetic Effect

The nutritional and medicinal plant *M. oleifera* has elevated certain concerns because of its prospective usage in the management of diabetes mellitus. Type 2 diabetes, particularly, is a long‐standing metabolic disorder that includes the regulation of glucose and insulin sensitivity. The therapeutic actions of *M. oleifera* are accredited to its bioactive constituents, and numerous investigations have engrossed its antidiabetic activities. These include vitamins, alkaloids, polyphenols, and flavonoids; collectively, they control blood sugar levels and augment the sensitivity to insulin (He et al. 2024). Here, we are going to deliberate the mechanisms by which *M. oleifera* modulates glucose metabolism and insulin sensitivity. By augmenting glucose metabolism, *M. oleifera* mostly mitigates diabetes. Certain of the plant bioactive compounds that are vital in plummeting blood glucose levels comprise chlorogenic acid and quercetin. These compounds have been revealed to inhibit the carbohydrate hydrolyzing enzymes α‐glucosidase and α‐amylase from generating glucose. The mode of action of moringa is to constrain these enzymes which inhibit postprandial upsurges in blood glucose, which assists in managing diabetes efficiently (Wang et al. 2022). *M. oleifera* also affects the activity of proteins that play a noteworthy role in glucose utilization and metabolism. As per Hamza et al. (2023), the bioactive compounds in moringa can augment glucose uptake by increasing insulin receptor expression on cells predominantly in skeletal muscle and adipose tissue. Improving overall glucose regulation by overcoming insulin resistance a—distinguishing characteristic of type 2 diabetes has—this imperative side effect. Insulin resistance, one of the main reasons for diabetes, can be exacerbated by *M. oleifera* . Through heightened sensitivity of cells to insulin, moringa diminishes the level of blood glucose and improves its uptake. The fertility of the plant with antioxidant flavonoids and polyphenolic phytochemicals is in part responsible for this effect. The insulin receptor signaling is disturbed by oxidative stress; however, the free radical scavenging effect of moringa can make cells more receptive to insulin (Mthiyane et al. 2022). Moringa potentiates the action of pancreatic β‐cells that are critical to insulin secretion. There is certain evidence; Aja et al. (2015) have stated, that moringa extracts can restore pancreatic cells which would upsurge insulin output. This is most critical in the early phases of diabetes, when pancreatic function is compromised by elevated blood glucose chronically. Major contributors to the onset and progression of diabetes are inflammation and oxidative damage. Chronic oxidative impairment and inflammation inhibit insulin signaling and β‐cell function, resulting in more adverse consequences for diabetic patients. Diminishing these effects will be meaningfully helped by the antioxidant properties of *M. oleifera*.

The plant delimited high levels of antioxidants, including vitamin C, β‐carotene, and polyphenol that decrease the concentrations of oxidative impairment to cells and tissues, as recognized by Fatoumata et al. (2020). Improving insulin sensitivity and glucose control, oxidative stress is abridged due to oxidative status decreasing, thereby dropping systemic inflammation. Moringa has been described to decrease pro‐inflammatory cytokine levels, including TNF‐α and IL‐6 in diabetics. Through the reserve of inflammation and enhancement of insulin receptor stimulation, moringa increases the effectiveness of insulin activity and glucose metabolism (Mthiyane et al. 2022). Numerous studies in animal and human models have recognized that *M. oleifera* contains antidiabetic activity. Studies on diabetic mice have revealed that moringa extracts are capable of decreasing blood glucose levels. In one study, Moringa leaf extract was initiated to meaningfully lessen blood glucose levels and improve insulin sensitivity in rats that had been induced to diabetes by alloxan (Aja et al. 2015). Supplementation with Moringa leaf powder or extract suggestively decreases fasting blood glucose, as has been exposed in human clinical trials. It suggests that it can be a functional diet for diabetes management, as Hamza et al. (2023) designates. In addition, moringa's actions are not only limited to blood glucose administration but also extend beyond antidiabetic drugs. The capability of moringa to augment glucose metabolism and lower diabetes complications like kidney injury, retinopathy, and neuropathy also adds to its therapeutic potential (Wang et al. 2022). Its numerous action mechanisms of augmenting insulin secretion, plummeting oxidative stress, improving insulin sensitivity, and refining glucose uptake make *M. oleifera* a fantastic natural drug for treating diabetes. Subsequently, it has a number of bioactive compounds that synergistically control blood sugar levels; this plant is helpful in stopping and treating diabetes. Experts and physicians are only just discovering the frequent health benefits of moringa, which holds potential for the diets, traditional medications, and general health of diabetic patients.

#### 3.1.5 Antimicrobial Effect

With its immune, ornamental, and antimicrobial possessions, the medicinal plant *M. oleifera* is a functional food contender. Moringa owns antiviral and antibacterial activities and can augment immune system function, as per investigation. The antimicrobial activity is due to bioactive composites like flavonoids, alkaloids, and phenolic acids, which hold antiviral and antimicrobial activities (Tariq et al. 2023). As per Mehwish et al. (2022), moringa is proficient in modifying immune reactions as it increases cytokine generation and the level of activity between immune cells such as macrophages and lymphocytes. To put it presently, this immunomodulating effect strengthens and defends the immune system in contradiction to pathogens (Ibrahim 2020). Secondly, moringa is present to be immunoenhancing by numerous models of experiments. For instance, as stated by El Shanawany et al. (2019), the immune system of naturally infested animals can be improved using its aqueous extract, which is immunomodulatory. The results demonstrate that the plant is proficient in improving immunity to both infectious and noninfectious illnesses. Choukse et al. (2022) and Frazzoli et al. (2023) discovered that moringa, a natural supplement, might be a feasible option for immunity development and infection deterrence.

#### 3.1.6 Neuroprotective Effect

A nutritious plant with medical solicitations is the “drumstick tree” or *M. oleifera* . A body of investigation increasingly recommends that it has neuroprotective properties specifically for cognitive function and neurodegenerative disease. It is theorized that moringa's neurodegenerative, anti‐inflammatory and antioxidant possessions are capable to constrain or slow neurological complaints such as Alzheimer's and Parkinson's disease, as well as other cognitive damages. Systematic studies have exposed that the bioactive composites in moringa comprising vitamins, polyphenols and flavonoids own a neuroprotective effect. For example, flavonoids are defensive of neurons from oxidative stress and inflammation, two principal inducers of neurodegenerative illnesses, owing to their antioxidant and anti‐inflammatory possessions. It has been established through scientific investigation that moringa extracts reduce the fabrication of reactive oxygen species (ROS). These ROS cause neurodegenerative complaints such as Alzheimer's and Parkinson's (González‐Burgos et al. 2021). Besides, critical signaling pathways linked with neuroinflammation and neurodegeneration are reduced by moringa. The neuroprotective actions of moringa are directly associated with the signaling pathways such as NF‐κB, Nrf2, and HO‐1. Mundkar et al. (2022) research indicates that moringa is a known neuroinflammation reducer, a signature of many neurodegenerative illnesses, by hindering the NF‐κB pathway, which has been implicated in inflammation. Srivastava and Ganjewala (2024) report that antioxidant reactions are controlled by the Nrf2 transcription factor, which is stimulated by moringa and therefore guards neurons against oxidative impairment.

Moringa has been stated to exert an influence on cognitive function in both animal studies and human studies. Cognitive function, learning, and memory can be improved by moringa extracts, particularly when cognitive impairment or oxidative stress is present. For instance, studies concerning rats have revealed that the amnestic drug scopolamine can be overturned by supplementing with moringa (Arozal et al. 2022). The hippocampus is an imperative brain area for memory and learning, and it is likely that moringa exerts this advantage through enhancing its function. Moringa can slow down or even avert the progression of neurological diseases such as Alzheimer's because of its neuroprotective properties. Moringa could help recover neurite outgrowth and the existence of neurons based on Hannan et al. (2014). Such procedures are critical to upholding brain function and reducing neurodegenerative injury.

These potentials are vital to identifying and treating neurodegenerative diseases such as Alzheimer's which bring about large neuronal death and synaptic connection loss. Moringa can be medicinally used to cure neurodegenerative illnesses due to its neuroprotective action. Oxidative stress, neuro‐inflammations, and the accumulation of misfolded proteins defining Alzheimer's and Parkinson's disease might just take reprieve from moringa supplementation. For illustration, in neurodegenerative sickness models in animals, moringa extracts with high antioxidant and anti‐inflammatory activities. These properties lower the injury to dopaminergic neurons in Parkinson's disease and avert amyloid plaques from adding in Alzheimer's disease, as per investigation (Ghimire et al. 2021; Srivastava and Ganjewala 2024). Studies have exposed that moringa is able to protect brain cells against damage by stopping amyloid‐beta plaque formation which is characteristic of Alzheimer's (Kim et al. 2022). In the same way, moringa antioxidant properties slow down the pace of disease progression and recover motor skills in Parkinson's disease through refurbishment of oxidative impairment to dopaminergic neurons (Hassan et al. 2021).

#### 3.1.7 Skin Health

Conventional medicine has used moringa for a long time for its anti‐inflammatory, antioxidant, and antibacterial properties in the skin. Moringa provides healthy skin due to its high content of vitamin A, vitamin C, and essential fatty acids. A shortage of these nutrients causes damage to the skin, oxidative stress, and accelerated aging. Free radicals that make skin age are proficiently neutralized by the antioxidants present in moringa. The manufacture of collagen in the skin is reinforced by the beta‐carotene and vitamin C present in the moringa leaf extract, which makes the skin charming and wrinkle‐free (Chhikara et al. 2021). In addition, the antioxidants present in moringa defend the skin against ultraviolet radiation, which, in turn, contributes to accelerating skin aging and causes an upsurge in the risk of skin cancer. Psoriasis, eczema, and acne are only some of the skin conditions that can be facilitated by moringa's anti‐inflammatory effect. Moringa tree extracts comprise anti‐inflammatory and anti‐reddening effects, making it a good remedy for skin irritation and healing (Gopalakrishnan et al. 2016). Furthermore, the antibacterial effect of moringa makes it an outstanding remedy for bacterial infections like acne because its ingredients abolish bad bacteria on the skin.

#### 3.1.8 Gastrointestinal Health

The advantageous effects of Moringa on digestive health have also been renowned. The deterrence of various GI diseases and gut health relies on the antibacterial and anti‐inflammatory properties. A further benefit to digestive health is the improved intestinal motility and normal bowel movements due to the high fiber content of the plant. Studies have demonstrated that moringa can also relieve a host of gastrointestinal symptoms such as gas, bloating, and constipation. Plant bioactive compounds activate the secretion of digestive enzymes which improve the digestion of food and augment nutrient uptake. Besides, moringa's anti‐ulcer properties have been studied. Certain investigations have indicated that the herb can reduce the severity of gastric ulcers by lowering the inflammatory and oxidative stress factors liable for their formation (Mehwish et al. 2022). Moringa is rich in tannins and flavonoids which not only heal but also defend the stomach lining. In addition, Moringa comprises antibacterial compounds that ease the management of gastrointestinal conditions produced by harmful bacteria and diseases. According to Kashyap et al. (2022), antimicrobial complexes in moringa can be exploited to treat and ward off bacteria‐induced gastrointestinal infections such as those triggered by *Salmonella* and *E. coli* . For appropriate digestive health, there needs to be a balanced gut microbiota, and Moringa certifies this by endorsing the development of good bacteria while constraining the growth of harmful germs.

#### 3.1.9 Anticancer Effect

Probably among its more appealing aspects is *M. oleifera* 's potential in the form of an anticancer agent. Plenty of bioactive substances that make it promising also exhibited proven anticancer properties, which would involve polyphenols and flavonoids through inhibition, apoptosis (programmed death of the cells), etc., along with preventing cancerous cells from metastasis. It has been found that moringa suppresses the growth of many types of cancer cells. Studies have shown that Moringa extract, particularly from the seeds and leaves, can inhibit the growth of cancer cells by blocking several signaling pathways involved in cell division and survival. For example, it has been found that isothiocyanates in moringa, such as moringin, induce cell cycle arrest and death and significantly reduce the viability of cancer cells (Wu et al. 2021).

Additionally, one of the primary causes that result in cancer is the oxidative damage of DNA, which is prevented by the antioxidant compounds in moringa. Moringa is also helpful in preventing cancer in its early stages by changing the expression of genes involved in cancer development. The chemopreventive qualities of moringa are also affected by its ability to reduce inflammation, a significant risk factor for cancer development. Based on the research done by Tiloke et al. (2018), it has been seen that Moringa nanoparticles show great promise towards antiproliferative activity against various cancer cells; this may be useful as supplemental therapy in the treatment of cancers. More recently, it has been shown that moringa enhances the cytotoxicity of standard chemotherapeutic drugs on cancer cells but diminishes their side effects. Moringa extracts, for instance, have been proven in studies to strengthen the effectiveness of chemotherapy drugs such as doxorubicin and cisplatin and could, therefore, be considered an adjuvant in the treatment of cancer (Shahbaz et al. 2024). Beyond its neuroprotective capability, *M. oleifera* is a multipurpose plant with many other health benefits. Scientific study shows its anti‐inflammatory, anticancer, anti‐microbial, and antioxidant properties, which support its use in gastrointestinal health, skin health, and cancer prevention. Nature Moringa comprises bioactive constituents that possess substitute functions, like cancer anticipation, ornamental digestion, and skin well‐being. Its prospective and standardized medical usages' development can be fully valued with further investigation and clinical trials (Table 4).

**TABLE 4:** Health benefits of *Moringa oleifera* .

Health benefit	Key findings	References
Antioxidant effects	Moringa leaf peptides showed significant antioxidant properties, combating oxidative stress	Avilés‐Gaxiola et al. (2021)
Anti‐inflammatory activity	Crude extracts reduced inflammation in lipopolysaccharide‐stimulated microglial cells	Mairuae et al. (2023)
Cardiovascular health	Extracts demonstrated cholesterol‐lowering and vascular protective effects	Alia et al. (2022)
Antidiabetic properties	Improved glucose regulation and insulin sensitivity in diabetic models	Mthiyane et al. (2022)
Immune system boost	Enhanced immune responses and antimicrobial activity in animal studies	Xiao et al. (2020)
Neuroprotective potential	Moringa extracts alleviated oxidative‐stress‐induced neurotoxicity in neuronal cells	Hashim et al. (2021), Islam et al. (2021)
Anti‐cancer activity	Moringa seed oil inhibited tumor cell proliferation	Shahbaz et al. (2024)
Skin health benefits	Topical application reduced skin inflammation and hyperproliferation	Cretella et al. (2020)
Gastrointestinal health	Anti‐inflammatory effects improved gut health in rodent models	Camilleri and Blundell (2024)
Cognitive function	Improved memory and reduced neuroinflammation in scopolamine‐induced mice	Arozal et al. (2022)
Antihypertensive effects	Seeds showed protective effects against hypertension in rats	Randriamboavonjy et al. (2016)
Immune‐boosting properties	Moringa supplementation enhanced poultry immune health	Mahfuz and Piao (2019)
Anti‐cancer mechanisms	Phytonanoparticles derived from *Moringa oleifera* showed antiproliferative activity against cancer cells	Tiloke et al. (2018)
Liver protection	Extracts improved liver function indices in diabetic rats	Aja et al. (2015)
Neurodegenerative diseases	Protective effects in Alzheimer's and Parkinson's models, with mitochondrial regulation	Ghimire et al. (2021)

## 4 Functional Properties of *Moringa oleifera*

**FIGURE 4:**
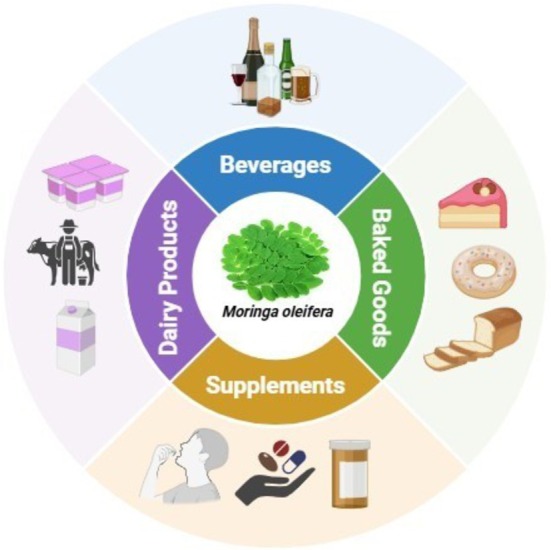
Utilization of *Moringa oleifera* .

Functional food and beverage progress is bringing *M. oleifera* , a plant with recognized nutritional and therapeutic actions, into the limelight. Moringa has been combined in frequent health foods such as powder, supplements, herbal tea, energy drinks, and fortified juice due to the dense content of bioactive chemicals, minerals, vitamins, and antioxidants. What follows is the discussion of the solicitation of moringa in diverse forms comprising health drinks, powders, capsules, herbal teas, energy drinks, and fortified juices (Figure 4).

### 4.1 *Moringa oleifera* Leaf Powder and Its Application in Functional Foods

An active component in food products, *M. oleifera* leaf powder (MOLP) has enhanced functional properties (Mohamed et al. 2022). It progresses the texture and mouthfeel of foods due to its outstanding water and oil absorbing capability (Fidyasari et al. 2024). It is also appropriate for use in dairy, beverage, and bread products because of its emulsifying and foaming properties, which upsurge product consistency (Singh and Prasad 2013). The antioxidant and anti‐inflammatory properties of fortified foods are improved by the presence of bioactive compounds like polyphenols, flavonoids, and isothiocyanates (Islam et al. 2021). However, MOLP has some shortcomings that can disturb consumer acceptability, such as a bright green color, bitter taste, and grassy flavor (Chan et al. 2021). Debittering and amalgamation with complementary constituents are necessary to improve palatability as a consequence of these sensory problems (Abdelwanis et al. 2024).

### 4.2 Impact on Food Characteristics

Bread, cookies, soups, and infant complementary nourishments are certain of the functional foods that have positively incorporated MOLP (Boateng et al. 2019; Sengev et al. 2013). Studies have designated that MOLP improves food products' overall nutritional quality by enhancing their protein, fiber, and micronutrient levels (Biel et al. 2017; Olson et al. 2016). Yet, the flavor, texture, color, and odor of food can all be affected by its occurrence. MOLP‐fortified wheat bread, for example, contained more protein but was thicker in texture and had a minor bitterness demanding formulation modifications to preserve consumer suitability, Sengev et al. (2013) reported. As described by Shiriki et al. (2015), MOLP‐enriched baby food presented the potential to decrease malnutrition but required optimal tuning to ensure palatability. Consumer approval of MOLP‐based functional foods is largely driven by influences such as component compatibility, processing approaches, and cultural dietary habits. Strategic formulation can improve marketability and customer attitude through the use of complementing flavors or covering substances, as per investigation (Zungu et al. 2020). In contrast to MOLP, which delivers a shelf‐stable, shelf‐ready substitute, fresh *M. oleifera* leaves deliver a superior nutrient composition, but their perishable nature limits their solicitation (Vergara‐Jimenez et al. 2017). On balance, MOLP holds much potential for the fabrication of nutrient‐rich functional foods if sensory concerns are sufficiently addressed to ensure consumer suitability and business feasibility.

Due to its frequent applications in food manufacture, agriculture, and industry, *M. oleifera* has been the topic of widespread studies. Of implication to the nutritional and medical solicitations of Moringa leaves is that their antioxidant activity seasonally differs in Pakistan, based on investigation (Iqbal and Bhanger 2006). In accumulation, biochemical investigation of Pakistani Moringa seed oil has produced good consequences for edible oil manufacture, as well as for industrial solicitations (Anwar and Bhanger 2003). Pakistani Moringa landraces are frugally appreciated, as they have the prospective to yield excellence oil (Faisal et al. 2020). Studies on the mineral conformation of Moringa leaves have also exposed that its nutritional worth is not continuous from region to region, which disturbs its usage in agriculture and foodstuff (Afzal et al. 2020).

Experts from Togo have examined the influence of a number of ecological influences on *M. oleifera* plant development and manufacture in agroforestry systems (Abotsi et al. 2015). The present investigation in Africa is exploring the nutritional and medicinal solicitations of Moringa and revisions have also exposed it to be vital for sustainable food security enterprises (El Bilali et al. 2024). An investigation study by N'nanle et al. (2020) exposed that the presence of Moringa leaves in broiler feed improved the performance, egg quality, and lipid metabolism of breeder hens. Moringa is an appreciated product in Pakistan and Togo, as the above studies disclose its multilayered usages in human nutrition, agriculture, animal husbandry, and industrial oil manufacture, among others.

#### 4.2.1 Powders and Supplements

These moringa powder and its byproducts have been popular due to the straightforward, nutrient‐dense supplement added to the regular diet. Most dried Moringa leaves contain healthy nutrients in the plant, making the goods from these. This is because Moringa powder is a rich source of proteins, vitamins including A, C, and E, minerals like calcium, potassium, and iron, and vital fatty acids supplementing general health and wellness. Moringa powders make various health drinks, capsules, and functional powders. These health drinks made from the moringa plant extract with a high antioxidant content are also commonly advertised as immune boosters, detoxifiers, and energy gainers. Due to claims of boosting digestion, adding vigor, reducing inflammation, and promoting healthy skin, moringa powders and capsules are sold as dietary supplements (Mehwish et al. 2022). The powders are also used in protein shakes, smoothies, and nutritional bars to meet the increasing demand for plant‐based, nutrient‐dense products. Sportspeople and fitness enthusiasts are reverting to moringa supplements for their natural vitamin load which assists in recovery and stamina. Due to its great bioavailability in a range of functional food forms, moringa has arisen in admiration as a food supplement (Mutar et al. 2021).

#### 4.2.2 Beverages

There is a great range of beverages that can be improved with moringa due to its versatility. Drinks such as these are not only delicious snacks but they also produce tangible advantages in the form of enhanced digestion, augmented antioxidant support, and a healthier immune system. We'll inspect three prominent groups of drinks that utilize moringa: energy drinks, herbal teas, and fortified juices below. The most predominant technique by which moringa has been rummage‐saled traditionally is as tea, yet its history of solicitation in herbal medicine has also been well documented. The minor earthy flavor and rich nourishment of this tea are an outcome of the moringa leaves that are rummage‐saled in its preparation. Its first usage is that of a beverage to increase immune function by virtue of comprising high levels of vitamin C as well as acting as an antioxidant (Pareek et al. 2023). To its detox, antimicrobial, and anti‐inflammatory actions, moringa also has renown of subsidiary healthy digestion and all‐around wellness. Moringa herbal teas likewise own functional effects; they are invigorating and have multipurpose usages. Aside from its own health compensations, moringa can be promoted in taste and harvested from allied health welfares when balancing with other herbs like ginger, mint, or lemon. Natural health practitioners are commencing to pay consideration to moringa herbal teas for its supposed potential to decrease fatigue, ease weight loss, and decrease blood sugar levels (Tshabalala et al. 2019). Growing admiration for hydrating functional drinks with extra health properties is fueling the presence of moringa in energy drinks. Moringa is a plant‐based stimulant as it has an assemblage of vitamins, minerals, and plant‐based proteins.

Vitamins B, C, and E are important for energy metabolism and cognitive function, so these drinks are typically formulated to enhance endurance, reduce fatigue, and improve cognitive functions (Abdelwanis et al. 2024). Moringa is often blended with other natural ingredients, such as ginseng, green tea extract, or caffeine, in the formulation of energy drinks to give an additional impetus without artificial stimulants. A practical component of sports nutrition and recovery drinks after exercise, moringa's antioxidant‐rich composition also mitigates the oxidative stress induced by extreme physical activity (Noaman et al. 2022). They also have more attractiveness and may interest customers seeking natural alternatives to conventional, highly caffeinated energy drinks. Moringa‐based fortified juices are gaining preference because they increase the nutrient value of fruit‐based beverages. Adding moringa powder or extract to fruit juices, such as orange, pomegranate, or apple, can result in more vitamins, minerals, and antioxidants. The moringa nutrients supplement the nutrients in fruits to give a healthy drink that augments digestion, immunity, and overall nutritional intake (Rodrigues et al. 2023). Customers also have moringa‐enriched juices available on shelves, which are sold as anti‐aging, anti‐inflammatory, and detox drinks. The beverages are gaining reputation among health‐aware consumers who are seeking to raise their intake of plant‐based nutrients. Certain producers of the beverages also provide them with additives such as probiotics or fiber to increase their health benefits even more (Perumalsamy et al. 2024). That *M. oleifera* is also functional in functional products like powders, health drinks, and capsules indicates how versatile it can be as a superfood. With increasing interest in functional foods and beverages, moringa has been getting increased attention for its outstanding nutritional profile and a number of prospective health benefits. Uses of moringa products vary from improved energy to improved skin health, and they are a natural, nutrient‐dense alternative to traditional dietary supplements and beverages. Increased research on moringa is bringing with it the potential for novel functional products that combine the healthful properties of the plant into a wider variety of consumer items. To augment the nutritional quality of beverages such as herbal teas, energy drinks, and juices, moringa is being exploited in functional food production on a large scale for generations to come.

#### 4.2.3 Baked Goods and Snacks

The “miracle tree,” or *M. oleifera* , is making its way into increasingly more food products due to its rich vitamin, mineral, and antioxidant profile. Energy bars, crackers, and biscuits are only a few of the innovative baked foods and snacks that can be enhanced by it. Adding protein, fiber, and essential micronutrients such as calcium, potassium, vitamins A and C, and moringa makes these foods even healthier. In addition, moringa's antioxidant potential associated with bioactive compounds such as polyphenols and flavonoids presents health benefits via free radical scavenging, potentially resulting in inflammation and oxidative stress reduction (Milla et al. 2021). The dietary quality of usual foods such as biscuits and crackers can be enhanced through the addition of moringa, based on research. For example, Cervera‐Chiner et al. (2024) researched the impact of powdered Moringa leaves incorporated into crackers as a snack. Not only did the authors enhance protein, fiber, and minerals considerably, but they also found that the sensory properties were acceptable. Energy bars, popular in the health and fitness market, also have powdered moringa leaf in them. Including the naturally occurring energy‐boosting moringa in a snack bar package provides consumers with what they desire: convenience and portability (Devisetti et al. 2016). Functional foods that have numerous health benefits and filling nutrition can be created by incorporating moringa into snacks.

In that respect, moringa stands as an ideal ingredient for fortified food since it improves metabolic health, among other benefits, and stimulates immune function (Madukwe et al. 2013). Such results indicate an increasing desire for functional snacks that are better for long‐term health and well‐being since they go beyond nutrient sufficiency.

#### 4.2.4 Dairy Products

*Moringa oleifera* is becoming increasingly accepted as a strengthening factor in the dairy and substitute dairy industry. Due to its richness in proteins and calcium, moringa can be used in a rich combination with milk and yogurt, which are rich dairy products. This addition makes dairy products nutritious but also answers the current trend in demand for functional meals containing more health benefits. An example of a good fortification would be the fortification of yogurt with moringa. It has been proven through research that Moringa leaf extract can enhance the nutritional content of yogurt in terms of protein, vitamins, and minerals without affecting its taste (Salem et al. 2013). Consumers also enjoy longer shelf life and stability in dairy products due to the antioxidant property of moringa. The addition of an aqueous moringa extract to yogurt enhanced its antioxidant activity, which may assist in combating oxidative stress, reported El‐Gammal et al. (2017). Moreover, plant‐based milk alternatives such as almond or soy may be nutritionally improved through the addition of moringa. Plant‐based drinks' functional ability and nutritional refilling may be boosted through the addition of moringa, reported Matabura and Rweyemamu (2022). Further health benefits are offered by the beverages because of the high antioxidant activity, which is supplemented with the rich polyphenol content in moringa (Rodrigues et al. 2023). Sour cream and other fermented dairy foods produced using moringa have been evaluated for the resulting health benefits. Besides dairy and plant‐based milk alternatives, it enhances the nutritional quality and microbiological stability (Salem et al. 2015). Natural, plant‐based, and functional diets are where it is at, and these trends will delight anyone seeking dairy products that enhance their well‐being.

The potential for *M. oleifera* in the functional food industry includes developing baked products, snack foods, and dairy/dairy substitute products. Being an intensive fortifier, enrichment for commodities, and potential health benefits via improving antioxidant activity, immunological support, and metabolic health are just several positive aspects of a well‐thought‐out nutrition and bioactive component profile. Due to many of its applications and benefits, moringa has become one of the significant contributors to the development of health‐conscious food products, for which demand has been increasing over the years for functional food among consumers.

## 5 Culinary Applications of *Moringa oleifera*

*Moringa oleifera* , or the “drumstick tree,” is known for its nutritional value and flexibility in diverse preparations. One of its numerous uses includes cooking soups, sauces, and ready‐to‐eat meals due to its nutrient composition comprising vitamins, minerals, and bioactive compounds (Madukwe et al. 2013). Moringa is an excellent nutrifier to enhance food nutrition because its leaves contain essential amino acids, antioxidants, and many vitamins such as calcium, A, and C.

### 5.1 Soups and Sauces

Moringa leaves and powders are often added to soups and sauces. These applications, where the leaves are added fresh or dried, are widespread in Asian, Latin American, and African cuisines. When added to soups, moringa provides a lot of nutrients that enhance flavor and offer health benefits. An example is the technique whereby the subtle heat of moringa adds depth to lentil and leafy green soups. Soups can be nutritionally fortified, and micronutrients can be combined into the diet with comfort in the high protein and antioxidant levels of moringa (Gautier et al. 2022). Moringa powder‐based sauces have even superior health benefits. The intense polyphenol content of moringa, including flavonoids and phenolic acids, can be practical as a natural preservative to extend the life of sauces without the addition of additional preservatives, as transcribed by Madukwe et al. (2013). Meanwhile, it is ironic that minerals are lost in plant‐based diets; at times, moringa is an outstanding supplement to plant‐based eaters' sauces.

### 5.2 Ready‐to‐Eat Meals

With the cumulative aspiration for speedy and healthy consumables, ready‐to‐eat (RTE) foodstuffs have gained noteworthy popularity among customers. With Moringa powder or extract in various RTE meal formulations added by producers such as rice meals, vegetable blends, and ready soups, moringa has found its specific niche in this category of food products. Persons who may not readily admit fresh vegetables may still enter minerals such as calcium and iron through these foods (Vanajakshi et al. 2015). The addition of Moringa powder in ready‐to‐consume meals improves their antioxidant functions, permitting them to be more effective in repelling oxidative stress, which can result in numerous long‐lasting diseases. In accordance with Badejo et al. (2014), the antioxidant activity of moringa is mainly attributed to its richness in polyphenols, such as chlorogenic acid and quercetin. Being a plant with the ability to augment the nutritional value of ready‐to‐eat foods, moringa is progressively being used in the food sector as a suitable yet nutrient‐dense functional food substitute appropriate for today's daily life (Table 5).

**TABLE 5:** Applications of *Moringa oleifera* in functional food products developments.

Application area	Examples	References
Moringa powders and supplements	Health drinks, capsules, and powders	Mehwish et al. (2022), Pareek et al. (2023)
Incorporation in beverages	Herbal teas, energy drinks, and fortified juices	Tshabalala et al. (2019), Matabura and Rweyemamu (2022)
Baked goods and snacks	Moringa‐enriched biscuits, crackers, and energy bars	Sengev et al. (2013), Cervera‐Chiner et al. (2024)
Dairy products	Moringa‐fortified yogurts and milk	Salem et al. (2013), El‐Gammal et al. (2017)
Plant‐based dairy alternatives	Fortified almond or oat milk with Moringa extracts	Gupta et al. (2018), Rodrigues et al. (2023)
Culinary applications	Soups, sauces, and ready‐to‐eat meals	Pop et al. (2022), Abdelwanis et al. (2024)
By‐product utilization	Seeds, pods, and leaves are used to create sustainable products	Gautier et al. (2022), Falowo et al. (2018)
Probiotic beverages	Moringa and beetroot‐based functional drinks	Vanajakshi et al. (2015), Trigo et al. (2023)
Nutritional fortification	Wheat flour or bread enriched with Moringa leaf powder	Rabie et al. (2020), Khan et al. (2017)
Sensory‐enhanced snacks	Moringa cookies and crackers with improved taste profiles	Devisetti et al. (2016), Oppusunggu et al. (2023)
Functional beverages	Fortified juice blends and antioxidant beverages	Noaman et al. (2022), Bailey‐Shaw et al. (2021)
Child nutrition products	Fortified complementary foods to reduce anemia	Shija et al. (2019), Hadju et al. (2020)
Maternal nutrition	Supplements to improve maternal health and birth weight	Hadju et al. (2020), Oyeyinka and Oyeyinka (2018)
Animal nutrition	Poultry and livestock feed enriched with Moringa	Mahfuz and Piao (2019), Adesina et al. (2013)
Cosmetic applications	Moringa seed oil for skincare and haircare products	Chen et al. (2022), Tshingani et al. (2017)

## 6 Challenges and Limitations of *Moringa oleifera* (MO) in Functional Foods and Medicinal Applications

Despite the several nutritional and medicinal assistances of *Moringa oleifera* (MO), numerous contests need to be overwhelmed before it can be applied in functional foods and medicine. Essential amino acid content restriction, low solubility of MO leaf precipitate, bitter palate, and unwanted color in cooked meals are just certain of these contests. To improve the adequacy and effectiveness of MO‐based solutions, these glitches need to be addressed. Essential amino acid EAAs establish an important portion of MO leaves, which are rich in protein. However, certain EAAs are found in fewer than optimum quantities, and the EAA profile of MO is not generally balanced (Olson et al. 2016). This restriction can disturb the nutritional quality of foodstuffs made from MO, chiefly if they are used as the main source of protein. A better amino acid profile can be attained by combining MO with other protein foods such as grains or legumes (Biel et al. 2017).

One of the serious glitches in food formulation is the poor water and other solvent solubility of MO leaf powder. This bounds its solicitation in beverages and other liquid‐based functional nourishments (Fidyasari et al. 2024). To improve the dispersibility and bioavailability of MO leaf powder, methods such as microencapsulation, nanoemulsification, and the usage of solubility refining substances have been discovered (Kashyap et al. 2022). In accumulation, the solubility of bioactive composites can be heightened by improving extraction approaches comprising selective pressured hot water extraction (Nuapia et al. 2020).

The penetrating flavor and bitter taste of MO leaves are main customer deterrence issues in food products chiefly (Chan et al. 2021). Debittering actions such as blanching, fermentation, and the usage of masking substances have been employed to alleviate these sensory consequences. For illustration, Chan et al. (2021) confirmed that blanching MO leaves to eradicate bitterness augmented the acceptability of instant soups fortified with MO meaningfully. In a similar way, including other elements, such as fruits or spices into MO can decrease its bitterness (Zungu et al. 2020).

The green color of MO leaf powder can take away from the aesthetic demand of some food dishes (Fidyasari et al. 2024). To offset this, food experts have come up with color masking procedures such as through the usage of natural pigments or through the usage of MO extracts low in chlorophyll content. Smoothies and green tea, which do not present a problem with their green color, can be complemented with MO, and this can make them further prevalent (Sengev et al. 2013). One of the chief inspirations for medical solicitation is the bioavailability of MO‐based bioactive composites. Manufacture and storage stability may recover the therapeutic activity of the bioactive substances (Chhikara et al. 2021).

It has been established that the bioactive constituents present in MO leaves are reserved by developing extraction technologies such as supercritical fluid extraction and ultrasound‐assisted extraction (Olvera‐Aguirre et al. 2022). In addition, encapsulation technologies have the capability to improve the bioavailability of these substances and defend them from deprivation (Perumalsamy et al. 2024).

Numerous approaches have been placed to pledge these contests. The permanence and solubility of *M. oleifera* leaf powder can be meaningfully improved by exploiting processing practices such as freeze‐drying and spray‐drying (Ariani et al. 2023). Customer acceptability is extremely reliant on sensory improvement, and the amalgamation of *M. oleifera* into acquainted food matrices such as bread, cookies, and soups has exposed potential in the part of flavor improvement (Rabie et al. 2020). In accumulation, debittering and flavor masking techniques can improve the taste of moringa‐based foodstuffs, creating them additional marketable (Chan et al. 2021). The nutritional deficits of Moringa can be appropriately overwhelmed at the same time the overall quality of fortified foods is enhanced via fortification and amalgamation with other nutrient‐dense constituents (Boateng et al. 2019). Furthermore, Moringa bioactive complexes' constancy, bioavailability, and controlled release may be improved via encapsulation and nanoformulations, predominantly for functional food and medicinal usages (Pop et al. 2022). In summary, although *M. oleifera* is a very auspicious functional food and medicinal constituent, it is also significant to tackle its drawbacks through groundbreaking formulation and processing approaches. With these hurdles overwhelmed, MO can be efficiently combined into many products for the assistances of improved health and nutrition.

## 7 Conclusion and Future Trends

*Moringa oleifera* is a nutrient‐dense, flexible plant with many health benefits; hence, its incorporation into functional meals will be perfect. It forms a rich diet for preventing oxidative stress, inflammation, and chronic diseases like diabetes and cardiovascular disorders owing to its content of essential vitamins, minerals, fiber, and bioactive substances. Moringa might be used for a lot more than just cooking but can include it in baked foods, snacks, drinks, dairy items, and more to enhance them with a healthier improvement. A number of matters, such as sensory characteristics, steadiness of bioactive substances, and scalability, need to be discussed before moringa can be extensively rummaged in the food industry. In spite of these contests, the worldwide market is prepared for moringa's appearance, particularly seeing the cumulative popularity of ecologically friendly, health‐focused, and plant‐based foodstuffs. In conclusion, moringa would have an energetic role in the functional food industry, and this would be beneficial for both producers and customers. The frequently emerging nature of food science and technology is optimistic for the functional food usages of *M. oleifera* . The bioactive composites in moringa are envisioned to be more efficiently extracted and formulated by applying novel methods such as nanoencapsulation to improve their bioavailability and stability in processed foods. Moringa can become prevalent among customers in most parts of the biosphere when enhancements in food processing technology purify it and make it non‐bitter and palatable. Moreover, moringa holds the stimulating capability to be combined with other healthy foods in a way that augments the total health benefits of the goods via cooperation. Moringa can be mixed with other superfoods such as chia, turmeric, or spirulina in the preparations of these product lines.

This role of moringa in contemporary health can be protractedly auxiliary with personalized nutrition that varies the nutritional recommendations based on an individual's needs. Customized nutrition plans incorporating moringa may result in customers experiencing health benefits tailored to their dietary requirements. In the value‐added use of moringa byproducts such as seeds and pods, further chances are given for the bettering environmental and financial sustainability of the moringa sector and sustainable farming. With all these applications, such as bioplastics, animal feed, and beauty products, a more sustainable and profitable industry can be achieved using byproducts. There is a bright future for products with base moringa functional food, which is full of chances for innovative, sustainable production and market growth despite the obstacles posed by factors that seem hard to overcome. Global implementation of moringa in functional foods would be spurred by further studies on harnessing its full health benefit, solving challenges in mass production, and developing easy‐to‐consumer products.

## Author Contributions

**Muhammad Tayyab Arshad:** methodology (equal), writing – original draft (equal). **Sammra Maqsood:** data curation (equal), writing – review and editing (equal). **Ali Ikram:** supervision (equal), validation (equal). **Kodjo Théodore Gnedeka:** project administration (equal), visualization (equal).

## Disclosure

Institutional Review Board Statement: The authors have nothing to report.

## Consent

The authors have nothing to report.

## Conflicts of Interest

The authors declare no conflicts of interest.

## Data Availability

The data supporting this study's findings are available from the corresponding author upon reasonable request.
